# Circ-METTL15 stimulates the aggressive behaviors of papillary thyroid cancer cells by coordinating the miR-200c-3p/XIAP axis

**DOI:** 10.55730/1300-0152.2689

**Published:** 2023-12-07

**Authors:** YuHao HUANG, XinYu ZENG, YanLing CAI, Yan YANG, YuJie ZHANG, YiQi MA, SuPing LI

**Affiliations:** 1 Department of Nuclear Medicine, The Affiliated Hospital of North Sichuan Medicine College, Nanchong City, Sichuan Province, China

**Keywords:** Papillary thyroid cancer, circ-METTL15, miR-200c-3p/XIAP, apoptotic

## Abstract

**Background/aim:**

Papillary thyroid carcinoma (PTC) is the most common form of thyroid cancer. The critical importance of circular RNA (circRNA) in a range of cancer types has been lately recognized. However, research on the functions of circRNAs in PTC has been limited thus far. Therefore, this research aimed at exploring the function and mechanism of circ-methyltransferase-like 15 (METTL15) in PTC cells.

**Materials and methods:**

Quantitative measurements of circ-METTL15, miR-200c-3p, and X-linked inhibitor of apoptosis protein (XIAP) in PTC cells were conducted using reverse transcription-quantitative polymerase chain reaction or Western blot analysis. To investigate cell growth, cell counting kit-8 and colony formation tests were employed, apoptosis was analyzed using flow cytometry, and migration and invasion were studied through Transwell assays. The targeted binding sites between miR-200c-3p and circ-METTL15 or XIAP were predicted by starBase and then verified by dual luciferase reporter assay.

**Results:**

circ-METTL15 and XIAP were upregulated in the PTC cells, while miR-200c-3p was downregulated. Downregulating circ-METTL15 or upregulating miR-200c-3p resulted in inhibited proliferation, migration, and invasion of PTC cells, while promoting apoptosis. miR-200c-3p was the downstream molecule of circ-METTL15, and XIAP was the direct target of miR-200c-3p. Forcing XIAP expression obstructed circ-METTL15 silencing to inhibit PTC cell activity.

**Conclusion:**

By coopting miR-200c-3p/XIAP, Circ-METTL15 stimulates aggressive behavior in PTC cells.

## 1. Introduction

Most thyroid cancers are papillary thyroid carcinomas (PTC), which are highly invasive and metastatic (Cabanillas et al., 2016). While the 5-year survival rate for PTC can reach 97%, some patients still experience early metastasis, chemotherapy resistance, and disease recurrence (Kunavisarut, 2013). At present, emerging evidence shows that noncoding RNAs (ncRNAs) are involved in PTC, especially circRNA with high stability, which is a promising diagnostic and prognostic biomarker (Tallini et al., 2015; Liyanarachchi et al., 2016; Xu and Jing, 2020). Hence, circRNA’s function and mechanism in PTC warrant further investigation.

Human genomes are largely composed of noncoding genes (Dhamija and Menon, 2018), and noncoding transcripts with circular structures, or circular RNAs, belong to the class of circular RNAs (circRNAs) (Chen and Yang, 2015; Kristensen et al., 2019). Studies have increasingly documented the role circRNAs play in physiological and pathological processes, such as alternative splicing (Ashwal-Fluss et al., 2014), microRNA (miRNA) sponge (Thomson and Dinger, 2016), and protein-RNA interactions and gene expression (Ebbesen et al., 2016). Various eukaryotes contain circular RNAs that are implicated in disease pathogenesis (Beermann et al., 2016; Han et al., 2018). In human malignancies, circRNAs are dysregulated in an increasing number (Meng et al., 2017; Zhang et al., 2017; Li et al., 2020; Beilerli et al., 2022). PTC progression is regulated by circRNAs, including cell proliferation and glycolysis (Liu et al., 2020; Xu and Jing, 2020). For example, circRUNX1 accelerates tumor metastasis through regulation of cell proliferation and invasion (Chu et al., 2021). Circular NEURL4 can regulate miR-1278 to target LATS1 and therefore inhibit PTC cell proliferation and invasion (Ding et al., 2021). Circular methyltransferase-like 15 (circ-METTL15) is a circRNA derived from METTL15. circ-METTL15 is overexpressed in lung cancer cells and tissues, and silencing it reduces lung cancer malignancy (Zhang et al., 2022). Nevertheless, the function of circ-METTL15 in PTC has not yet been elucidated.

As miRNA sponges, circRNAs can regulate tumor progression (Hansen et al., 2013; Wang et al., 2016; Beilerli et al., 2022). A significant role has been demonstrated for miRNAs in cancer development (Tan et al., 2018). Through bioinformatics website analysis, it was found that miR-200c-3p has a potential binding site for circ-METTL15. miR-200c-3p inhibits the progression of breast, ovarian, and liver cancers (Zhang et al., 2019; Anastasiadou et al., 2021; de la Cruz-Ojeda et al., 2022). This study examined the targeting relationship between circ-METTL15 and miR-200c-3p in PTC cells and explored their functional correlation in PTC. As miRNAs bind to target mRNAs in the 3’untranslated region (3′UTR), they regulate gene expression posttranscriptionally (Fabian et al., 2010). The targeting relationship between miR-200c-3p and X-linked inhibitor of apoptosis protein (XIAP) was predicted using the bioinformatics website. A member of the IAP family, XIAP inhibits cell death by blocking the downstream pathways of apoptosis (Tu and Costa, 2020; Hanifeh and Ataei, 2022). XIAP has been reported to be upregulated in many human malignancies (Holcik et al., 2001), including PTC, and is correlated with the invasive phenotype (Xiao et al., 2007; Yim et al., 2011). The presence of miRNA binding sites or reaction elements in circRNAs enables them to reduce miRNA expression and inhibits their ability to regulate target genes at transcriptional or posttranscriptional levels (Hansen et al., 2013; Jarlstad Olesen and Kristensen, 2021). At the same time, circRNA/miRNA/mRNA axes have been identified in PTC progression (Li et al., 2021).

Therefore, the current study speculated that circ-METTL15 may compete with miR-200c-3p to modulate XIAP and further affect PTC malignant progression. To test this speculation, molecular biology techniques and bioinformatics methods were employed, eventually illustrating the interaction of circ-METTL15, miR-200c-3p, and XIAP in PTC.

## 2. Methods

### 2.1. Clinical sample

Forty paired PTC and adjacent tissues were collected from patients at the Affiliated Hospital of North Sichuan Medicine College, promptly preserved at −80 °C to prevent RNA depletion, and verified by pathologists. Radiotherapy and chemotherapy were not administered to any of the patients prior to surgery. The Ethics Committee of the Affiliated Hospital of North Sichuan Medicine College issued an approval document for the human study, and all of the patients signed informed consent forms.

### 2.2. Cell culture

The culture cells including normal thyroid cells (Nthy-ori3-1) and PTC cell lines SW1736, TPC-1, and SW579 (all from the Cell Bank of Chinese Academy of Sciences, Shanghai, China) were treated with Roswell Park Memorial Institute (RPMI-1640) medium, which included 10% fetal bovine serum (FBS) and 1% penicillin-streptomycin. A cell incubator was employed with the appropriate conditions set (37 °C, 5% CO_2_).

### 2.3. Cell transfection

Lipofectamine 2000 was used (Thermo Fisher Scientific Inc., Waltham, MA, USA) for transfection performed on the TPC-1 cells. The plasmids (GenePharma, Shanghai, China) included sh-METTL15, sh-NC, oe-METTL15, oe-NC, miR-200c-3p mimic, mimic NC, miR-200c-3p inhibitor, inhibitor NC, sh-METTL15 + oe-XIAP, and sh-METTL15 + oe-NC.

### 2.4. Cell counting kit-8 (CCK-8) test

TPC-1 cells were inoculated into 96-well plates, with each well containing 2 × 10^3^ cells. After 72 h, 10 μL of CCK-8 solution (Beijing Solarbio Science & Technology Co., Ltd., Beijing, China) was added to each well. Four hours after the reaction, the absorbance level at 450 nm was measured using a microplate reader (Thermo-Fisher Scientific Inc.).

### 2.5. Colony formation analysis

Cell colonies were formed in 6-well plates, with each well initially containing 200 TPC-1 cells. Two weeks later, the observable colonies were stabilized using methanol and colored with 0.1% crystal violet (Sigma-Aldrich Chemical Co., St. Louis, MO, USA), and those consisting of ≥50 cells were counted under a microscope (Olympus Scientific Solutions, Shinjuku, Tokyo, Japan).

### 2.6. Flow cytometry

Apoptotic measurements were conducted using an Annexin V-FITC apoptosis detection kit (Beijing Solarbio Science & Technology Co.). TPC-1 cells were configured into a cell suspension with a binding buffer and mixed with propidium iodide solution to achieve 100 μg/mL. After 10 min, the suspension was loaded onto the flow cytometer (BD Biosciences, San Jose, CA, USA) and analyzed using FlowJo software.

### 2.7. Transwell assays

Diluted Matrigel solution (Sigma-Aldrich Chemical Co.) was covered in the upper chamber to analyze cell invasion but not cell migration. TPC-1 cells suspended in 100 μL of serum-free medium were inoculated into the upper compartment and infiltrated into the lower compartment containing 10% FBS-RPMI-1640 medium (500 μL) for 24 h. With mechanical removal of noninvasive cells, the infiltrated cells were fixed with methanol, stained with crystal violet (Sangon Biotech, Shanghai, China), and counted under a light microscope (Olympus Scientific Solutions).

### 2.8. Reverse transcription-quantitative polymerase chain reaction (RT-qPCR)

RNA was harvested from PTC tissues and cell lines using Trizol reagents (Thermo Fisher Scientific Inc.) and then subjected to reverse transcription. The TaqMan reverse transcription kit (Thermo Fisher Scientific Inc.) was used for circ-METTL15 or XIAP, while the miRNA first strand cDNA synthesis kit (Tiangen, Beijing, China) was used for miR-200c-3p. For assessing RNA, the SYBR Premix Ex TaqIM II kit was utilized for PCR analysis (Takara Bio Inc., Kusatsu, Shiga, Japan), and the 2^−ΔΔCt^ method was employed to measure target gene expression, which was normalized to U6 or glyceraldehyde-3-phosphatedehydrogenase (GAPDH). The primer sequences are demonstrated in Table 1.

### 2.9. Western blot analysis

Tissue and cells underwent lysis in radioimmunoprecipitation assay buffer (Beyotime Biotechnology, Shanghai, China) and concentration analysis using a bicinchoninic acid assay kit (Bio-Rad, Hercules, CA, USA). The protein (30 μg/lane) was separated by 12% sodium dodecyl sulfate polyacrylamide gel electrophoresis and electrotransferred to a polyvinylidene fluoride membrane (Bio-Rad). After 1-h blockade with 5% skim milk at 37 °C, primary antibody XIAP (2042) or GAPDH (2118; both from Cell Signaling Technology, Danvers, MA, USA) was mixed overnight at 4 °C and horseradish peroxidase-conjugated secondary antibody (Abcam, Waltham, MA, USA) for 1 h. The blots, made visible through enhanced chemiluminescence (Cell Signaling Technology), were analyzed using Image Lab software (V4.0; Bio-Rad).

### 2.10. Dual luciferase reporter (DLR) gene assay

The StarBase database (http://starbase.sysu.edu.cn/) was used to explore circ-METTL15 and miR-200-c-3p possible targets. Wild-type and mutant reporters for circ-METTL15 or XIAP 3’UTR containing predicted binding sequences for miR-200c-3p were designed (Promega Corp., Madison WI, USA) and cotreated with miR-200c-3p mimic or mimic NC into TPC-1 cells, respectively. Luciferase activities were determined at 48 h posttransfection using a DLR system (Promega Corp.).

### 2.11. Data statistics

Every piece of data underwent processing via IBM SPSS Statistics for Windows 21.0 (IBM Corp., Armonk, NY, USA), with measurement data presented as mean ± standard deviation, adhering to a normal distribution pattern. Two groups were compared using Student’s t-test, while multiple groups were compared using one-way analysis of variance and Tukey’s test. Correlation analysis of the clinical samples was performed using the Pearson method. It was accepted that a p < 0.05 was statistically significant.

## 3. Results

### 3.1. Forced circ-METTL15 expression in the PTC cells

**Figure 1:**
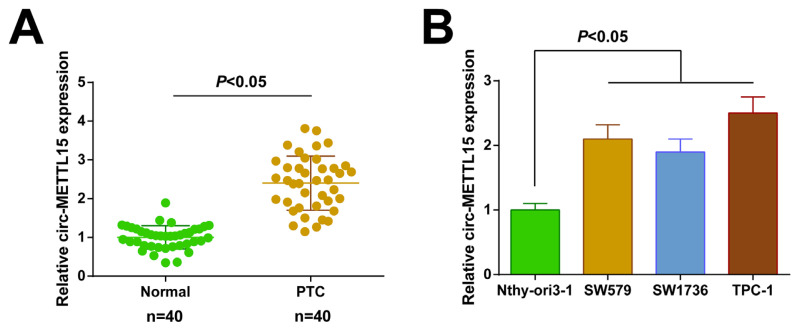
Upregulated expression of circ-METTL15 in PTC. A) RT-qPCR to check circ-METTL15 in PTC and normal tissues; B) RT-qPCR to check circ-METTL15 in PTC cell lines. All of the data are measurement data, and the values are expressed as the mean ± standard deviation.

The RT-qPCR results revealed that the circ-METTL15 in PTC tissues was significantly forced (Figure 1A). The clinical information table indicated that circ-METTL15 was related to the tumor size, tumor, node, and metastasis (TNM) stage, and N stage (Table 2). The circ-METTL15 expression trend in the PTC cell lines (SW1736, TPC-1, and SW579) was upward, particularly in the TPC-1 cells (Figure 1B). Therefore, the TPC-1 cells became the study object for the cellular experiments.

### 3.2 Aggressive PTC cell behavior downregulated by circ-METTL15

**Figure 2:**
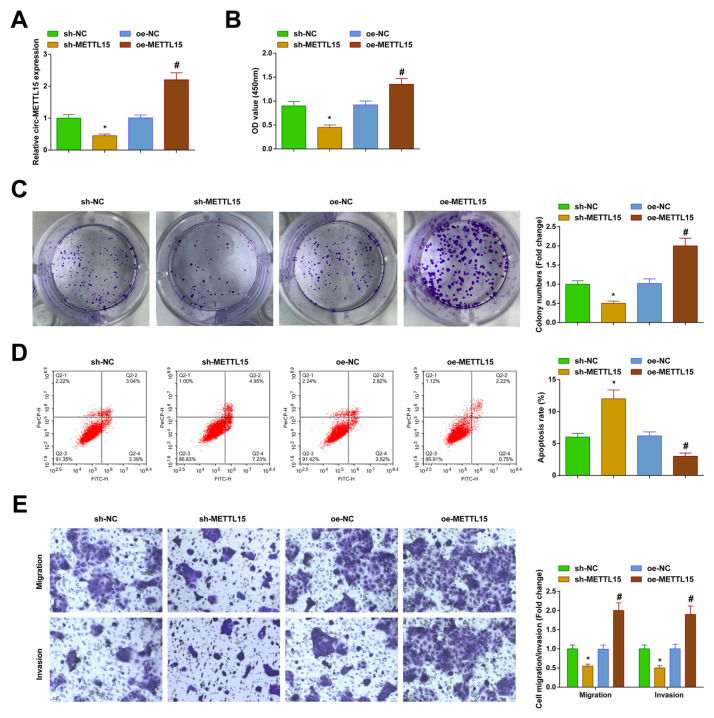
Downregulated circ-METTL15 suppresses PTC cell growth. A) RT-qPCR to verify the transfection; B-C) CCK-8 and colony formation assays to measure cell proliferation; D) flow cytometry to analyze apoptosis; E) Transwell assays to detect cell migration and invasion. All of the data are measurement data, and the values are expressed as the mean ± standard deviation. * p < 0.05 vs. sh-NC; # p < 0.05 vs. oe-NC.

Circ-METTL15 low/high expression vectors (sh-METTL15/oe-METTL15) were investigated in the TPC-1 cells, and the RT-qPCR verified the transfection (Figure 2A). CCK-8 and clonogenesis results suggested that the proliferation of TPC-1 cells was reduced after the downregulation of circ-METTL15 (Figures 2B and 2C). Using flow cytometry, apoptotic measurements determined that apoptosis of the TPC-1 cells enhanced after the downregulation of circ-METTL15 (Figure 2D). Transwell assay findings revealed that circ-METTL15 downregulation reduced the number of migrating and invading TPC-1 cells (Figure 2E). In contrast, upregulated circ-METTL15 led to the opposite result (Figures 2B–2E).

### 3.3. miR-200c-3p is the downstream molecule of circ-METTL15

**Figure 3:**
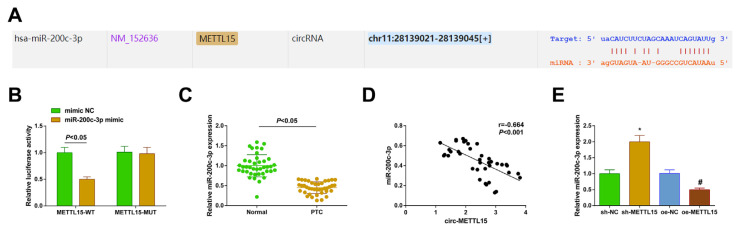
miR-200c-3p is the downstream molecule of circ-METTL15. A) Target binding site of circ-METTL15 and miR-200c-3p; B) DLR assay to verify the targeting relationship between circ-METTL15 and miR-200c-3p; C) RT-qPCR to check miR-200c-3p in PTC tissues and normal tissues; D) correlation between circ-METTL15 and miR-200c-3p; E) RT-qPCR to check miR-200c-3p after the intervention of circ-METTL15. All of the data are measurement data, and the values are expressed as the mean ± standard deviation. * p < 0.05 vs. sh-NC; # p < 0.05 vs. oe-NC.

The tool StarBase 3.0 was employed to identify possible specific binding sites between circ-METTL15 and miR-200c-3p (Figure 3A). A DLR assay was performed to discover if miR-200c-3p mimic reduced the luciferase activity of the METTL15-WT (Figure 3B). Data analysis revealed downregulation of miR-200c-3p in the clinical samples and indicated a negative correlation with circ-METTL15 expression (Figures 3C and 3D). Finally, the RT-qPCR analyses revealed miR-200c-3p in the TPC-1 cells, which interfered with the circ-METTL15 expression, and it was demonstrated that the miR-200c-3p expression was elevated after the downregulation of circ-METTL15. After upregulating the circ-METTL15, the miR-200c-3p level was suppressed (Figure 3E).

### 3.4. PTC cell malignancy constrained by upregulated miR-200c-3p

**Figure 4:**
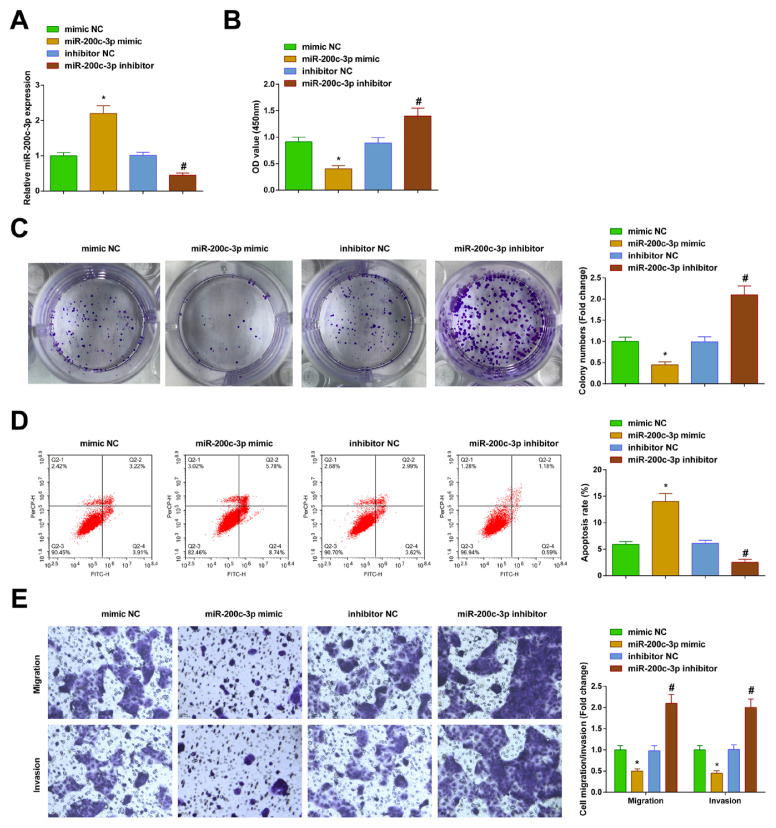
Upregulating miR-200c-3p inhibits PTC cell development. A) RT-qPCR to verify the transfection; B–C) CCK-8 and colony formation assays to measure cell proliferation; D) flow cytometry to analyze apoptosis; E) Transwell assays to detect cell migration and invasion. All of the data are measurement data, and the values are expressed as the mean ± standard deviation. * p < 0.05 vs. mimic NC; # p < 0.05 vs. inhibitor NC.

The miR-200c-3p mimic and inhibitor were transfected into the TPC-1 cells, respectively. Successful transfection was verified by RT-qPCR (Figure 4A). Various experimental results indicated that upregulation of miR-200c-3p weakened the proliferative, migratory, invasive, and antiapoptotic abilities, whereas downregulation of miR-200c-3p strengthened these abilities (Figures 4B–4E).

### 3.5. XIAP is directly targeted by miR-200c-3p

**Figure 5:**
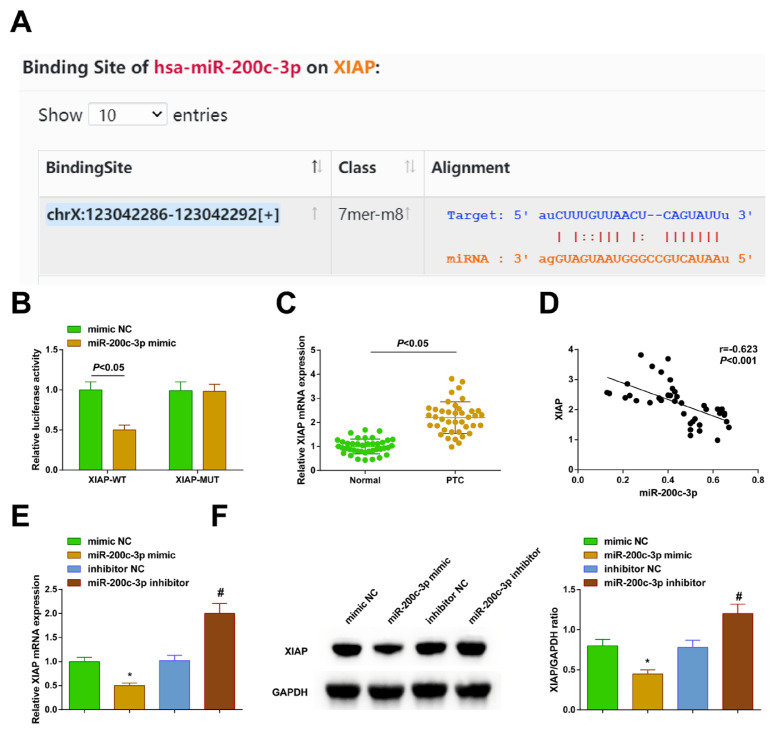
XIAP is the direct target of miR-200c-3p. A) Target binding site of miR-200c-3p and XIAP; B) DLR assay to verify the targeting relationship between miR-200c-3p and XIAP; C) RT-qPCR to check XIAP mRNA expression in PTC tissues and normal tissues; D) correlation between miR-200c-3p and XIAP mRNA expression; E) RT-qPCR and Western blot to measure XIAP after the intervention of miR-200c-3p. All of the data are measurement data, and the values are expressed as the mean ± standard deviation. * p < 0.05 vs. mimic NC; # p < 0.05 vs. inhibitor NC.

Using StarBase 3.0, the binding sites connecting miR-200c-3p and XIAP were identified (Figure 5A). Subsequently, the DLR assay revealed that miR-200c-3p mimic reduced the luciferase activity of the XIAP-WT reporter (Figure 5B). The XIAP mRNA expression was upregulated in the clinical samples and negatively correlated with the miR-200c-3p expression (Figures 5C and 5D). RT-qPCR and Western blot analyses supported that the XIAP in the TPC-1 cells interfered with miR-200c-3p, and there was a decrease after the upregulation of miR-200c-3p and an increase after downregulation (Figure 5E).

### 3.6. PTC cell aggressive behavior stimulates circ-METTL15 through coordinating the miR-200c-3p/XIAP axis

**Figure 6:**
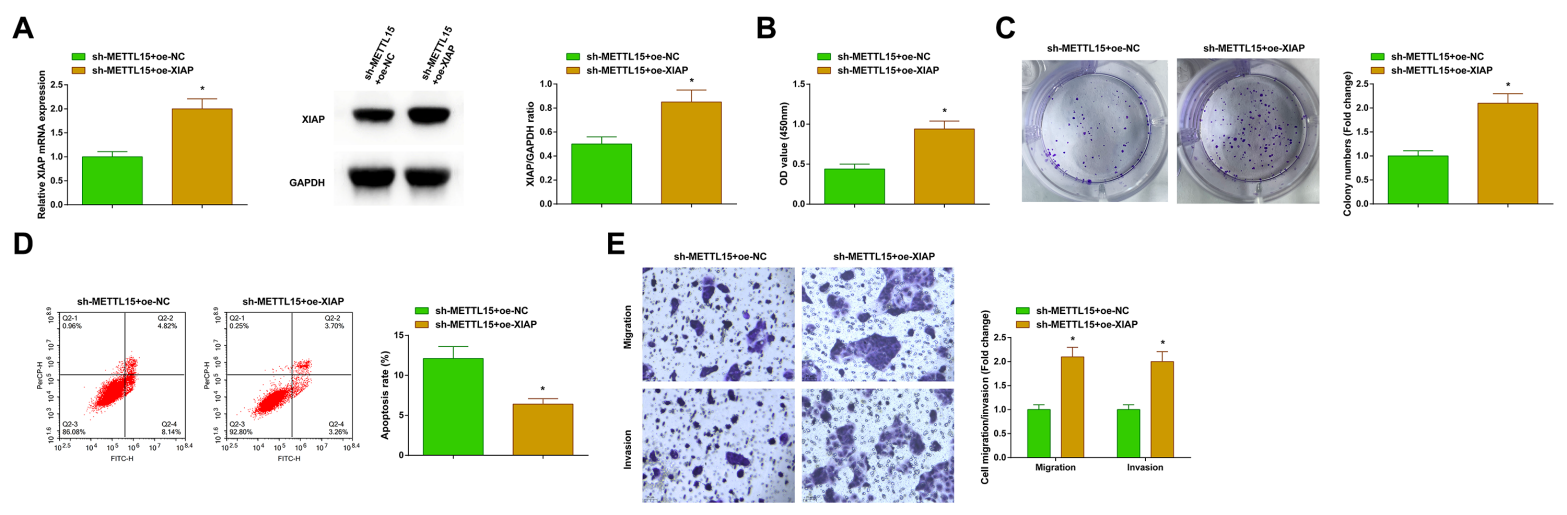
Circ-METTL15 stimulates the aggressive behaviors of PTC cells by coordinating the miR-200c-3p/XIAP axis. A) RT-qPCR and Western blot to verify the transfection; B–C) CCK-8 and colony formation assays to measure cell proliferation; D) flow cytometry to analyze apoptosis; E) Transwell assays to detect cell migration and invasion. All of the data are measurement data, and the values are expressed as the mean ± standard deviation. * p < 0.05 vs. sh-METTL15 + oe-NC.

sh-METTL15 + oe-XIAP were interfered with in the TPC-1 cells, and RT-qPCR and Western blot detected XIAP upregulation successfully (Figure 6A). Considering the anti-PTC effects of downregulated circ-METTL15, XIAP upregulation led to a reversal, contributing to the retrieval of TPC-1 cell malignancy (Figures 6B–6E).

## 4. Discussion

RNA sequencing technology and bioinformatics methods have facilitated the discovery of PTC-related circRNAs (Lan et al., 2018; Guo et al., 2020; Lv et al., 2021). CircRNAs, as oncogenes or tumor suppressors, are associated with onset and progression of cancer (Ma et al., 2018). For example, hsa_circ_0058124 enhances PTC tumor invasion via the NOTCH3/GATAD2A pathway (Yao et al., 2019). In addition, circRNAs with differential expressions may be reliable markers for the diagnosis of PTC (Shi et al., 2020). This study demonstrated that circ-METTL15 promoted the malignant behavior of the PTC cells by inducing XIAP expression through miR-200c-3p. Additionally, this research was the inaugural discovery of miR-200c-3p’s targeting of the circ-METTL15 or XIAP.

At present, very little research has been done on circ-METTL15. A study by Zhang et al. (2022) alone confirmed that circ-METTL15 expression in lung cancer is abundant, while silencing circ-METTL15 is a cancer-promoting factor by modulation of the invasion, proliferation, and immune escape. Based on this, the current study first detected circ-METTL15 in PTC and reported an abnormality in its high expression. Moreover, the high expression of circ-METTL15 was related to the tumor size, TNM stage, and N stage. Subsequently, functional assays proved that circ-METTL15 silencing impaired the malignancy of cancer cells, while circ-METTL15 overexpression stimulated PTC malignant progression, hinting that circ-METTL15 could not only be a promising diagnostic biomarker but also a therapeutic target for PTC.

Growing research indicates that circRNAs may act as miRNA sponges, leading to the enhanced expression of downstream genes (Bak and Mikkelsen, 2014; Panda, 2018). As an example, circ_0004458 aids in PTC by increasing RAC1 expression through the absorption of miR-885-5p (Jin et al., 2018). Circ-PSD3 enhances the malignant actions of PTC cells by increasing HEMGN levels through the absorption of miR-637 (Li et al., 2021). circ-METTL15 targeted miR-200c-3p in PTC cells. MiR-200c-3p demonstrated its ability to suppress tumors in multiple cancer types, PTC included (Liu et al., 2019; Posch et al., 2021; Liu et al., 2022). The current study found the downregulated miR-200c-3p in PTC. Considering its function, miR-200c-3p overexpression obstructed the malignant behavior of the cancer cells, while miR-200c-3p silencing had the opposite effect.

MiRNAs inhibit translation or trigger the degradation of specific mRNAs by attaching to their 3’UTR (de la Cruz-Ojeda et al., 2022). Predictions about the downstream targets of miR-200c-3p were made through the starBase bioinformatics website. The DLR gene assay verified that miR-200c-3p targets XIAP in the PTC cells. It should be noted that XIAP is highly expressed in anaplastic thyroid carcinoma and affects cancer cell activities (Liu et al., 2017). Moreover, Hussain et al. (2015) confirmed that XIAP is elevated in PTC and targeting XIAP is effective in managing PTC tumor progression. The present research found an increase in XIAP expression within the PTC tissues and cells. Elevated levels of miR-200c-3p may lead to a decrease in XIAP expression in PTC cells. Rescue experiments revealed that interference with circ-METTL15 somewhat curtailed the malignancy of PTC cells by diminishing XIAP expression. Circ-METTL15 played a role in enhancing XIAP expression by serving as a sponge for miR-200c-3p in PTC cells.

As for the limitations of this study, the interactions between XIAP and other protein factors were not explored to determine if they influence the malignant behavior of PTC. Therefore, in subsequent studies, the sample needs to be expanded to investigate the relationship between circ-METTL15 and the clinicopathological features and prognosis of PTC patients. Further molecular and animal experiments are required to explore how circ-METTL15 mediates the miR-200c-3p/XIAP axis to stimulate PTC malignancy.

## 5. Conclusion

Circ-METTL15 is overexpressed in PTC and correlates with the clinicopathological features of patients, suggesting its potential as a biomarker for clinical diagnosis. In addition, circ-METTL15 has a sponge-like effect on miR-200c-3p and regulates PTC development through XIAP, providing a new strategy for the clinical treatment of PTC

## Data availability

Data supporting the findings of this study are available from the corresponding author upon reasonable request.

## Figures and Tables

**Table 1:** Primer sequences.

Genes	Sequences
circ-METTL15	Forward: 5′- GCCAGCATCGTTGCAGATTT-3′
	Reverse: 5′- AGTTGCATGGAGGAACACCC-3′
miR-200c-3p	Forward: 5′- CGCTAATACTGCCGGGTAAT-3′
	Reverse: 5′- GCAGGGTCCGAGGTATTC-3′
XIAP	Forward: 5′- TGATCGTGCCTGGTCAGAAC-3′
	Reverse: 5′- GGTCTTCACTGGGCTTCCAA-3′
U6	Forward: 5′-CTCGCTTCGGCAGCACA-3′
	Reverse: 5′-AACGCTTCACGAATTTGCGT-3′
GAPDH	Forward: 5′-CACCCACTCCTCCACCTTTG-3′
	Reverse: 5′-CCACCACCCTGTTGCTGTAG-3′

**Table 2:** Correlation between the circ-METTL15 gene and clinical features in the patients with PTC.

Characteristics	Number	circ-METTL15		p-value
		High (n = 20)	Low (n = 20)	
Age (years)				1
≥45	19	9	10	
<45	21	11	10	
Sex				0.748
Male	24	11	13	
Female	16	9	7	
Tumor size (cm)				<0.001
≥2	13	12	1	
<2	27	8	19	
TNM stage				0.001
I–II	28	9	19	
III–IV	12	11	1	
N stage				0.01
N0	23	7	16	
N1	17	13	4	
